# Some generalizations of Hermite–Hadamard type inequalities

**DOI:** 10.1186/s40064-016-3301-3

**Published:** 2016-09-26

**Authors:** M. Rostamian Delavar, M. De La Sen

**Affiliations:** 1 Department of Mathematics, Faculty of Basic Sciences, University of Bojnord, Bojnord, Iran; 2 Institute of Research and Development of Processes, University of Basque Country, Campus of Leioa (Bizkaia) - Aptdo. 644- Bilbao, 48080 Bilbao, Spain

**Keywords:** $\eta$-convex function, Integral inequalities, Hermite–Hadamard inequality, 26A51, 26D15, 52A01

## Abstract

Some generalizations and refinements of Hermite–Hadamard type inequalities related to $\eta$-convex functions are investigated. Also applications for trapezoid and mid-point type inequalities are given.

## Introduction and preliminaries

This paper generalizes some well-known results for Hermite–Hadamard integral inequality by generalizing the convex function factor of the integrand to be an $\eta$-convex function. The obtained results have as particular cases those previously obtained for convex functions in the integrand.

The following inequality is well known in the literature as the Hermite–Hadamard integral inequality (Pecaric et al. 1991):

1 $$f\left( {\frac{a + b}{2}} \right) \le \frac{1}{b - a}\mathop \int \nolimits_{a}^{b} f\left( x \right)dx \le \frac{f\left( a \right) + f\left( b \right)}{2}.$$

where $f:\left[ {a,b} \right] \to {\mathbb{R}}$ be a convex function. For more results about (1), see Alomari et al. (2010), Dragomir (1992), Kirmaci (2004), Pearce and Pecaric (2000), Rostamian Delavar and Dragomir (2016), Rostamian Delavar et al. (to appear), Wasowicz and Witkowski (2012), Yang (2001), Yang et al. (2004) and references therein.

Let $I$ be an interval in real line ${\mathbb{R}}$. Consider $\eta :A \times A \to B$ for appropriate $A,B \subseteq {\mathbb{R}}$.

### **Definition 1** (Gordji et al. 2016)

A function $f:I \to {\mathbb{R}}$ is called convex with respect to $\eta$ (briefly $\eta$-convex), if

2 $$f\left( {tx + \left( {1 - t} \right)y} \right)\,\le\,f\left( y \right) + t\eta \left( {f\left( x \right),\,f\left( y \right)} \right),$$

for all $x,y \in I$ and $t \in \left[ {0,1} \right]$.

In fact above definition geometrically says that if a function is $\eta$-convex on $I$, then its graph between any $x,y \in I$ is on or under the path starting from $\left( {y,f\left( y \right)} \right)$ and ending at $(x,f\left( y \right) + \eta \left( {f\left( x \right),f\left( y \right)} \right)$. If $f\left( x \right)$ should be the end point of the path for every $x,y \in I$, then we have $\eta \left( {x,y} \right) = x - y$ and the function reduces to a convex one.

There exists $\eta$-convex functions for some bifunctions $\eta$ that are not convex. We have the following simple examples:

### *Example 2* (Gordji et al. 2015)

a. Consider a function $f:{\mathbb{R}} \to {\mathbb{R}}$ defined by

$$f\left( x \right) = \left\{ {\begin{array}{*{20}l} { - x,} \hfill &\quad {x \ge 0;} \hfill \\ {x,} \hfill &\quad {x < 0.} \hfill \\ \end{array} } \right.$$

and define a bifunction $\eta$ as $\eta \left( {x,y} \right) = - x - y$, for all $x,y \in {\mathbb{R}}^{ - } = \left( { - \infty ,0} \right].$ It is not hard to check that $f$ is a $\eta$-convex function but not a convex one.

b. Define the function $f:{\mathbb{R}}^{ + } \to {\mathbb{R}}^{ + }$ by

$$f\left( x \right) = \left\{ {\begin{array}{*{20}l} {x,} \hfill &\quad {0 \le x \le 1;} \hfill \\ {1,} \hfill &\quad {x > 1.} \hfill \\ \end{array} } \right.$$

and define the bifunction $\eta :{\mathbb{R}}^{ + } \times {\mathbb{R}}^{ + } \to {\mathbb{R}}^{ + }$ by

$$\eta \left( {x,y} \right) = \left\{ {\begin{array}{*{20}l} {x + y,} \hfill &\quad {x \le y;} \hfill \\ {2\left( {x + y} \right),} \hfill &\quad {x > y.} \hfill \\ \end{array} } \right.$$

Then $f$ is $\eta$-convex but is not convex.

The following theorem is an important result:

### **Theorem 3**

(Gordji et al. 2016) *Suppose that*$f:I \to {\mathbb{R}}$*is a*$\eta$-*convex function and*$\eta$*is bounded from above on*$f\left( I \right) \times f\left( I \right)$*. Then*$f$*satisfies a Lipschitz condition on any closed interval*$\left[ {a,b} \right]$*contained in the interior*$I^{ \circ }$*of*$I$*. Hence,*$f$*is absolutely continuous on*$\left[ {a,b} \right]$*and continuous on*$I^{ \circ }$.

### *Remark 4*

As a consequence of Theorem 3, an $\eta$-convex function $f:\left[ {a,b} \right] \to {\mathbb{R}}$ where $\eta$ is bounded from above on $f\left( {\left[ {a,b} \right]} \right) \times f\left( {\left[ {a,b} \right]} \right)$ is integrable.

The following simple lemma is required.

### **Lemma 5**

*Suppose that*$a,b \in {\mathbb{R}}$*. Then*

(i) $$\hbox{min} \{ a,b\} \le \frac{a + b}{2}$$.

(ii) *if*$f$, $g$*are integrable on*$\left[ {a,b} \right]$*then,*$\mathop \int \limits_{a}^{b} \hbox{min} \{ f,g\} = \hbox{min} \left\{ {\mathop \int \nolimits_{a}^{b} f,\mathop \int \nolimits_{a}^{b} g} \right\}$.

### *Proof*

Assertions are consequence of this fact:

$$\hbox{min} \{ a,b\} = \frac{{a + b - \left| {a - b} \right|}}{2}.$$

□

We have a basic lemma:

### **Lemma 6**

*Let*$f:\left[ {a,b} \right] \to {\mathbb{R}}$*be a*$\eta$-*convex function. Then for any*$t \in \left[ {0,1} \right]$*we have the inequalities*

3 $$\begin{aligned} \frac{1}{2}\left[ {f\left( {ta + \left( {1 - t} \right)b} \right) + f\left( {\left( {1 - t} \right)a + tb} \right)} \right] & \le \hbox{min} \left\{ {f\left( b \right) + \frac{1}{2}\eta \left( {f\left( a \right),f\left( b \right)} \right),f\left( a \right) + \frac{1}{2}\eta \left( {f\left( b \right),f\left( a \right)} \right)} \right\} \\ & \le \frac{1}{2}\left[ {f\left( a \right) + f\left( b \right)} \right] + \frac{1}{4}\left[ {\eta \left( {f\left( a \right),f\left( b \right)} \right) + \eta \left( {f\left( b \right),f\left( a \right)} \right)} \right], \\ \end{aligned}$$

4 $$\frac{1}{2}\left[ {f\left( {ta + \left( {1 - t} \right)b} \right) + f\left( {\left( {1 - t} \right)a + tb} \right)} \right] \le \frac{1}{2}\left[ {f\left( a \right) + f\left( b \right)} \right] + t\frac{1}{2}\left[ {\eta \left( {f\left( a \right),f\left( b \right)} \right) + \eta \left( {f\left( b \right),f\left( a \right)} \right)} \right],$$

5 $$f({ta + ( {1 - t} )b} ) \le \frac{1}{2}[ {f(a) + f(b)] + {\frac{1}{2}} [t\eta ( {f(a),f(b)}) + ({1 - t})\eta ( {f( b ),f( a )} )} ]$$

*and*

6 $$\begin{aligned} f\left( {\frac{a + b}{2}} \right) & \le \hbox{min} \left\{ f\left( {ta + \left( {1 - t} \right)b} \right) +\, \frac{1}{2}\eta \left( {f\left( {\left( {1 - t} \right)a + tb} \right),f\left( {ta + \left( {1 - t} \right)b} \right)} \right),f\left( {\left( {1 - t} \right)a + tb} \right) + \frac{1}{2}\eta \left( {f\left( {ta + \left( {1 - t} \right)b} \right),f\left( {\left( {1 - t} \right)a + tb} \right)} \right)\right\} \\ & \le \frac{1}{2}\left[ {f\left( {\left( {1 - t} \right)a + tb} \right) + f\left( {ta + \left( {1 - t} \right)b} \right)} \right] \\ & \quad + \frac{1}{4}\eta \left( {f\left( {\left( {1 - t} \right)a + tb} \right),f\left( {ta + \left( {1 - t} \right)b} \right)} \right) \\ & \quad + \frac{1}{4}\eta \left( {f\left( {ta + \left( {1 - t} \right)b} \right),f\left( {\left( {1 - t} \right)a + tb} \right)} \right). \\ \end{aligned}$$

### *Proof*

If in (2) we put $t$ instead of $1 - t$ and then add that inequality with (2) we have:

7 $$\frac{1}{2}\left[ {f\left( {ta + \left( {1 - t} \right)b} \right) + f\left( {\left( {1 - t} \right)a + tb} \right)} \right] \le f\left( b \right) + \frac{1}{2}\eta \left( {f\left( a \right),f\left( b \right)} \right)$$

for all $$t \in \left[ {0,1} \right].$$

If in (7) we replace $a$ with $b$ and add the result with (7), then we have (3).

Now, if in (2) we put $a$ instead of $b$ and then add that inequality with (2) we get:

$$f\left( {ta + \left( {1 - t} \right)b} \right) + f\left( {tb + \left( {1 - t} \right)a} \right) \le f\left( b \right) + f\left( a \right) + t\left[ {\eta \left( {f\left( a \right),f\left( b \right)} \right) + \eta \left( {f\left( b \right),f\left( a \right)} \right)} \right]$$

for all $t \in \left[ {0,1} \right],$ which is equivalent to (4).

If we change $a$ with $b$, and $t$ with $1 - t$ in (2) and then add that inequality with (2) we get:

$$2f\left( {ta + \left( {1 - t} \right)b} \right) \le f\left( b \right) + f\left( a \right) + t\eta \left( {f\left( a \right),f\left( b \right)} \right) + \left( {1 - t} \right)\eta \left( {f\left( b \right),f\left( a \right)} \right)$$

for all $t \in \left[ {0,1} \right]$ and the inequality (5) is proved.

Finally since we have

$$f\left( {\frac{a + b}{2}} \right) = f\left( {\frac{{ta + \left( {1 - t} \right)b + tb + \left( {1 - t} \right)a}}{2}} \right)$$

and

$$f\left( {\frac{a + b}{2}} \right) = f\left( {\frac{{tb + \left( {1 - t} \right)a + ta + \left( {1 - t} \right)b}}{2}} \right),$$

then by using (2) we can obtain (6) □

## Hermite–Hadamard type inequalities

In this section we obtain some Hermite–Hadamard type integral inequalities which improve right and left side of (1) respectively.

### **Theorem 1**

*Let*$f:\left[ {a,b} \right] \to {\mathbb{R}}$*be a*$\eta$-*convex function with*$\eta$*bounded from above on*$f\left( {\left[ {a,b} \right]} \right) \times f\left( {\left[ {a,b} \right]} \right)$*. Then we have inequalities*

8 $$\begin{aligned} \frac{1}{2}\mathop \int \limits_{0}^{1} [f\left( {ta + \left( {1 - t} \right)b} \right) + f\left( {\left( {1 - t} \right)a + tb} \right)] & \le \hbox{min} \left\{ {f\left( b \right) + \frac{1}{2}\eta \left( {f\left( a \right),f\left( b \right)} \right),f\left( a \right) + \frac{1}{2}\eta \left( {f\left( b \right),f\left( a \right)} \right)} \right\} \\ & \le \frac{1}{2}\left[ {f\left( a \right) + f\left( b \right)} \right] + \frac{1}{4}\left[ {\eta \left( {f\left( a \right),f\left( b \right)} \right) + \eta \left( {f\left( b \right),f\left( a \right)} \right)} \right], \\ \end{aligned}$$

9 $$\begin{aligned} \frac{1}{2}\mathop \int \nolimits_{0}^{1} [f\left( {ta + \left( {1 - t} \right)b} \right) + f\left( {\left( {1 - t} \right)a + tb} \right)] \le \frac{1}{2}\left[ {f\left( a \right) + f\left( b \right)} \right] + \frac{1}{2}\left[ {\eta \left( {f\left( a \right),f\left( b \right)} \right) + \eta \left( {f\left( b \right),f\left( a \right)} \right)} \right]\mathop \int \nolimits_{0}^{1} tdt \hfill \\ \hfill \\ \end{aligned}$$

*and*

10 $$\mathop \int \nolimits_{0}^{1} f\left( {ta + \left( {1 - t} \right)b} \right)dt \le \frac{1}{2}\left[ {f\left( a \right) + f\left( b \right)} \right] + \frac{1}{2}\eta \left( {f\left( a \right),f\left( b \right)} \right)\mathop \int \nolimits_{0}^{1} tdt + \frac{1}{2}\eta \left( {f\left( b \right),f\left( a \right)} \right)\mathop \int \nolimits_{0}^{1} \left( {1 - t} \right)dt.$$

### *Proof*

Since $\eta$ is bounded from above on $f\left( {\left[ {a,b} \right]} \right) \times f\left( {\left[ {a,b} \right]} \right)$, the note after Theorem 3, guarantees existence of above integrals. The inequalities (8)–(10) follow by Lemma 6 on integrating over $t \in \left[ {0,1} \right].$□

### *Remark 2*

If $f:\left[ {a,b} \right] \to {\mathbb{R}}$ is a $\eta$-convex function and $\eta$ is bounded from above on $f\left( {\left[ {a,b} \right]} \right) \times f\left( {\left[ {a,b} \right]} \right)$, then by Theorem 1 we have

11 $$\begin{aligned} \frac{1}{2}\mathop \int \nolimits_{a}^{b} [f\left( x \right) + f\left( {a + b - x} \right)]dx & \le \hbox{min} \left\{ {f\left( b \right) + \frac{1}{2}\eta \left( {f\left( a \right),f\left( b \right)} \right),f\left( a \right) + \frac{1}{2}\eta \left( {f\left( b \right),f\left( a \right)} \right)} \right\}\left( {b - a} \right) \\ & \le \frac{1}{2}\left[ {f\left( a \right) + f\left( b \right)} \right]\left( {b - a} \right) + \frac{1}{4}\left[ {\eta \left( {f\left( a \right),f\left( b \right)} \right) + \eta \left( {f\left( b \right),f\left( a \right)} \right)} \right]\left( {b - a} \right), \\ \end{aligned}$$

12 $$\frac{1}{2}\mathop \int \nolimits_{a}^{b} [f\left( x \right) + f\left( {a + b - x} \right)]dx \le \frac{1}{2}\left[ {f\left( a \right) + f\left( b \right)\left] {\left( {b - a} \right) + \frac{1}{2}} \right[\frac{{\eta \left( {f\left( a \right),f\left( b \right)} \right) + \eta \left( {f\left( b \right),f\left( a \right)} \right)}}{b - a}} \right]\mathop \int \nolimits_{a}^{b} \left( {x - a} \right)dx,$$

and

13 $$\mathop \int \nolimits_{a}^{b} f\left( x \right) \le \frac{1}{2}\left[ {f\left( a \right) + f\left( b \right)} \right]\left( {b - a} \right) + \frac{1}{2}\frac{{\eta \left( {f\left( a \right),f\left( b \right)} \right)}}{b - a}\mathop \int \nolimits_{a}^{b} \left( {x - a} \right)dx + \frac{1}{2}\frac{{\eta \left( {f\left( b \right),f\left( a \right)} \right)}}{b - a}\mathop \int \nolimits_{a}^{b} \left( {b - x} \right)dx.$$

All of inequalities (11)–(13) are different views for right side of generalized Hermite-Hadamard inequalities and finally can be stated as a unique form of

14 $$\frac{1}{b - a}\mathop \int \nolimits_{a}^{b} f\left( x \right)dx \le \frac{1}{2}\left[ {f\left( a \right) + f\left( b \right)} \right] + \frac{1}{4}\left[ {\eta \left( {f\left( a \right),f\left( b \right)} \right) + \eta \left( {f\left( b \right),f\left( a \right)} \right)} \right].$$

If we suppose that $\eta \left( {x,y} \right) = x - y$, then we recapture right side of (1).

Also we can obtain the following result:

### **Theorem 3**

*Let*$f:\left[ {a,b} \right] \to {\mathbb{R}}$*be a*$\eta$-*convex function with*$\eta$*bounded from above on*$f\left( {\left[ {a,b} \right]} \right) \times f\left( {\left[ {a,b} \right]} \right)$*. Then we have the inequalities:*

15 $$\begin{aligned} f\left( {\frac{a + b}{2}} \right) & \le \mathop \int \nolimits_{0}^{1} \hbox{min} \left\{ f\left( {ta + \left( {1 - t} \right)b} \right) + \frac{1}{2}\eta \left( {f\left( {\left( {1 - t} \right)a + tb} \right),f\left( {ta + \left( {1 - t} \right)b} \right)} \right),f\left( {\left( {1 - t} \right)a + tb} \right) + \frac{1}{2}\eta \left( {f\left( {ta + \left( {1 - t} \right)b} \right),f\left( {\left( {1 - t} \right)a + tb} \right)} \right)\right\} dt \\ & \le \hbox{min} \left\{ \mathop \int \nolimits_{0}^{1} f\left( {ta + \left( {1 - t} \right)b} \right)dt + \frac{1}{2}\mathop \int \nolimits_{0}^{1} \eta \left( {f\left( {\left( {1 - t} \right)a + tb} \right),f\left( {ta + \left( {1 - t} \right)b} \right)} \right)dt,\mathop \int \nolimits_{0}^{1} f\left( {\left( {1 - t} \right)a + tb} \right)dt + \frac{1}{2}\mathop \int \nolimits_{0}^{1} \eta \left( {f\left( {ta + \left( {1 - t} \right)b} \right),f\left( {\left( {1 - t} \right)a + tb} \right)} \right)dt\right\} \\ & \le \mathop \int \nolimits_{0}^{1} \frac{{f\left( {ta + \left( {1 - t} \right)b} \right) + f\left( {\left( {1 - t} \right)a + tb} \right)}}{2}dt + \frac{1}{4}\mathop \int \nolimits_{0}^{1} \left[\eta \left( {f\left( {\left( {1 - t} \right)a + tb} \right),f\left( {ta + \left( {1 - t} \right)b} \right)} \right) +\,\eta \left( {f\left( {ta + \left( {1 - t} \right)b} \right),f\left( {\left( {1 - t} \right)a + tb} \right)} \right)\right]dt. \\ \end{aligned}$$

### *Proof*

From (6) we have

$$\begin{aligned} f\left( {\frac{a + b}{2}} \right) \le \hbox{min} \left\{ f\left( {ta + \left( {1 - t} \right)b} \right) + \frac{1}{2}\eta \left( {f\left( {\left( {1 - t} \right)a + tb} \right),f\left( {ta + \left( {1 - t} \right)b} \right)} \right),f\left( {\left( {1 - t} \right)a + tb} \right) \quad +\,\frac{1}{2}\eta \left( {f\left( {ta + \left( {1 - t} \right)b} \right),f\left( {\left( {1 - t} \right)a + tb} \right)} \right)\right\} , \\ \end{aligned}$$

for any $t \in \left[ {0,1} \right].$ Integrating over $t$ we get the first inequality in (15). Now Using properties of Lemma 5 along with integrating rules gives

$$\begin{aligned}& \mathop \int \nolimits_{0}^{1} \hbox{min} \left\{ f\left( {ta + \left( {1 - t} \right)b} \right) + \frac{1}{2}\eta \left( {f\left( {\left( {1 - t} \right)a + tb} \right),f\left( {ta + \left( {1 - t} \right)b} \right)} \right), f\left( {\left( {1 - t} \right)a + tb} \right) + \frac{1}{2}\eta \left( {f\left( {ta + \left( {1 - t} \right)b} \right),f\left( {\left( {1 - t} \right)a + tb} \right)} \right)\right\} dt \hfill \\ &\le \hbox{min} \left \{ \mathop \int \nolimits_{0}^{1} f\left( {ta + \left( {1 - t} \right)b} \right)dt + \frac{1}{2}\mathop \int \nolimits_{0}^{1} \eta \left( {f\left( {\left( {1 - t} \right)a + tb} \right),f\left( {ta + \left( {1 - t} \right)b} \right)} \right)dt, \mathop \int \nolimits_{0}^{1} f\left( {\left( {1 - t} \right)a + tb} \right)dt + \frac{1}{2}\mathop \int \nolimits_{0}^{1} \eta \left( {f\left( {ta + \left( {1 - t} \right)b} \right),f\left( {\left( {1 - t} \right)a + tb} \right)} \right)dt\right\} \hfill \\& \le \mathop \int \nolimits_{0}^{1} \frac{{f\left( {ta + \left( {1 - t} \right)b} \right) + f\left( {\left( {1 - t} \right)a + tb} \right)}}{2}dt + \frac{1}{4}\mathop \int \nolimits_{0}^{1} \left[ {\eta \left( {f\left( {\left( {1 - t} \right)a + tb} \right),f\left( {ta + \left( {1 - t} \right)b} \right)} \right)} \right. \hfill \\& + \left. {\eta \left( {f\left( {ta + \left( {1 - t} \right)b} \right),f\left( {\left( {1 - t} \right)a + tb} \right)} \right)} \right]dt. \hfill \\ \end{aligned}$$

□

### *Remark 4*

If $f:\left[ {a,b} \right] \to {\mathbb{R}}$ is a $\eta$-convex function and $\eta$ is bounded from above on $f\left( {\left[ {a,b} \right]} \right) \times f\left( {\left[ {a,b} \right]} \right)$, then by Theorem 3 we have

16 $$\begin{aligned} f\left( {\frac{a + b}{2}} \right)\left( {b - a} \right) & \le \mathop \int \nolimits_{a}^{b} \hbox{min} \left\{ {f\left( {a + b - x} \right) + \frac{1}{2}\eta \left( {f\left( x \right),f\left( {a + b - x} \right)} \right),} \right.\left. {f\left( x \right) + \frac{1}{2}\eta \left( {f\left( {a + b - x} \right),f\left( x \right)} \right)} \right\}dx \\ & \le \hbox{min} \left\{ {\mathop \int \nolimits_{a}^{b} f\left( {a + b - x} \right)dx + \frac{1}{2}\mathop \int \nolimits_{a}^{b} \eta \left( {f\left( x \right),f\left( {a + b - x} \right)} \right)dx,} \right.\left. {\mathop \int \nolimits_{a}^{b} f\left( x \right)dx + \frac{1}{2}\mathop \int \nolimits_{a}^{b} \eta \left( {f\left( {a + b - x} \right),f\left( x \right)} \right)dx} \right\} \\ & \le \mathop \int \nolimits_{a}^{b} \frac{{f\left( {a + b - x} \right) + f\left( x \right)}}{2}dx + \frac{1}{4}\mathop \int \nolimits_{a}^{b} [\eta \left( {f\left( x \right),f\left( {a + b - x} \right)} \right) \\ & \quad + \eta \left( {f\left( {a + b - x} \right),f\left( x \right)} \right)]dx = \mathop \int \nolimits_{a}^{b} f\left( x \right)dx + \frac{1}{2}\mathop \int \nolimits_{a}^{b} \eta \left( {f\left( x \right),f\left( {a + b - x} \right)} \right)dx, \\ \end{aligned}$$

which gives a refinement for left side of (1). If we suppose that $\eta \left( {x,y} \right) = x - y$, then we recapture left side of (1).

## Trapezoid and mid-point type inequalities

An interesting question in (1), is estimating the difference between left and middle terms and between right and middle terms. In this section we investigate about this question, when the absolute value of the derivative of a function is $\eta$-convex. We need Lemma 2.1 in Kirmaci (2004):

### **Lemma 1**

*Suppose that*$f:\left[ {a,b} \right] \to {\mathbb{R}}$*is a differentiable mapping,*$g:\left[ {a,b} \right] \to {\mathbb{R}}^{ + }$*is a continuous mapping and*$f^{\prime}$*is integrable on*$\left[ {a,b} \right]$. *Then*

$$\frac{1}{b - a}\mathop \int \nolimits_{a}^{b} f\left( x \right)dx - f\left( {\frac{a + b}{2}} \right) = \left( {b - a} \right)\left[ {\mathop \int \nolimits_{0}^{1/2} tf^{\prime}\left( {ta + \left( {1 - t} \right)b} \right)dt + \mathop \int \nolimits_{1/2}^{1} (t - 1} )f^{\prime}\left( {ta + \left( {1 - t} \right)b} \right)dt\right],$$

### *Remark 2*

In Lemma 1, if we use the change of variable $x = tb + \left( {1 - t} \right)a$, then

$$\frac{1}{b - a}\mathop \int \nolimits_{a}^{b} f\left( x \right)dx - f\left( {\frac{a + b}{2}} \right) = \left( {b - a} \right)\left[ {\mathop \int \nolimits_{0}^{1/2} ( - t})f^{\prime}( {tb + ( {1 - t} )a} )dt + \mathop \int \nolimits_{1/2}^{1} (1 - t)f^{\prime}( {tb + ( {1 - t} )a} )dt\right],$$

Using Lemma 1, we can prove the following theorem to estimate the difference between the middle and left terms in (1).

### **Theorem 3**

*Suppose that*$f:\left[ {a,b} \right] \to {\mathbb{R}}$*is a differentiable mapping and*$\left| {f^{\prime}} \right|$*is an*$\eta$-*convex mapping on*$\left[ {a,b} \right]$*with a bounded*$\eta$*from above. Then*

$$\left| {\frac{1}{b - a}\mathop \int \nolimits_{a}^{b} f\left( x \right)dx - f\left( {\frac{a + b}{2}} \right)} \right| \le \frac{1}{8}\left( {b - a} \right)K,$$

*where*

$$K = \hbox{min} \left\{ \left| {f^{\prime}\left( b \right)} \right| + \frac{{\left| {\eta \left( {f^{\prime}\left( a \right),f^{\prime}\left( b \right)} \right)} \right|}}{2},\left| {f^{\prime}\left( a \right)} \right| + \frac{{\left| {\eta \left( {f^{\prime}\left( b \right),f^{\prime}\left( a \right)} \right)} \right|}}{2}\right\}$$

### *Proof*

From $\eta$-convexity of $\left| {f^{\prime}} \right|$, Theorem 3 and Lemma 1 it follows that

$$\begin{aligned} \left| {\frac{1}{b - a}\mathop \int \nolimits_{a}^{b} f\left( x \right)dx - f\left( {\frac{a + b}{2}} \right)} \right| & \le \left( {b - a} \right)\left\{ \mathop \int \nolimits_{0}^{1/2} t\left( {\left| {f^{\prime}\left( b \right)\left| { + t} \right|\eta \left( {f^{\prime}\left( a \right),f^{\prime}\left( b \right)} \right)} \right|} \right)dt + \mathop \int \nolimits_{1/2}^{1} (1 - t)\left( {\left| {f^{\prime}\left( b \right)\left| { + t} \right|\eta \left( {f^{\prime}\left( a \right),f^{\prime}\left( b \right)} \right)} \right|} \right)dt\right\} = \frac{1}{8}\left( {b - a} \right)\left[ {2\left| {f^{\prime}\left( b \right)\left| + \right|\eta \left( {f^{\prime}\left( a \right),f^{\prime}\left( b \right)} \right)} \right|} \right] = I \\ \end{aligned}$$

On the other hand according to Remark 2 we have

$$\begin{aligned} \left| {\frac{1}{b - a}\mathop \int \nolimits_{a}^{b} f\left( x \right)dx - f\left( {\frac{a + b}{2}} \right)} \right| & \le \left( {b - a} \right)\left\{ \mathop \int \nolimits_{0}^{1/2} ( - t)\left( {\left| {f^{\prime}\left( a \right)\left| { + t} \right|\eta \left( {f^{\prime}\left( b \right),f^{\prime}\left( a \right)} \right)} \right|} \right)dt + \mathop \int \nolimits_{1/2}^{1} (t - 1)\left( {\left| {f^{\prime}\left( a \right)\left| { + t} \right|\eta \left( {f^{\prime}\left( b \right),f^{\prime}\left( a \right)} \right)} \right|} \right)dt\right\} = \frac{1}{8}\left( {b - a} \right)\left[ {2\left| {f^{\prime}\left( a \right)\left| + \right|\eta \left( {f^{\prime}\left( b \right),f^{\prime}\left( a \right)} \right)} \right|} \right] = J \\ \end{aligned}$$

Then we can deduce the result from

$$\left| {\frac{1}{b - a}\mathop \int \nolimits_{a}^{b} f\left( x \right)dx - f\left( {\frac{a + b}{2}} \right)} \right| \le \hbox{min} \{ I,J\} .$$

□

### *Remark 4*

If in the proof of Theorem 3 we consider $\eta \left( {x,y} \right) = x - y$ for all $x,y \in \left[ {a,b} \right]$, we approach to Theorem 2.2 in Kirmaci (2004).

The following is Lemma 2.1 in Dragomir and Agarwal (1998).

### **Lemma 5**

*Suppose that*$f:\left[ {a,b} \right] \to {\mathbb{R}}$*is a differentiable function and*$f^{\prime}$*is an integrable function on*$\left[ {a,b} \right]$*. Then*

17 $$\frac{f\left( a \right) + f\left( b \right)}{2} - \frac{1}{b - a}\mathop \int \nolimits_{a}^{b} f\left( x \right)dx = \frac{1}{b - a}\mathop \int \nolimits_{a}^{b} (x - \frac{a + b}{2})f^{\prime}\left( x \right)dx.$$

*Using Lemma 5, we can prove the following theorem to estimate the difference between the middle and right terms in (*1*).*

### **Theorem 6**

*Suppose that*$f:\left[ {a,b} \right] \to {\mathbb{R}}$*is a differentiable function and*$\left| {f^{\prime}} \right|$*is an*$\eta$-*convex function where*$\eta$*is bounded from above on*$\left[ {a,b} \right]$*. Then*

$$\left| {\frac{f\left( a \right) + f\left( b \right)}{2} - \frac{1}{b - a}\mathop \int \nolimits_{a}^{b} f\left( x \right)dx} \right| \le \frac{1}{8}\left( {b - a} \right)K,$$

*where*

$$K = \hbox{min} \left\{ \left| {f^{\prime}\left( b \right)} \right| + \frac{{\left| {\eta \left( {f^{\prime}\left( a \right),f^{\prime}\left( b \right)} \right)} \right|}}{2},\left| {f^{\prime}\left( a \right)} \right| + \frac{{\left| {\eta \left( {f^{\prime}\left( b \right),f^{\prime}\left( a \right)} \right)} \right|}}{2}\right\} .$$

### *Proof*

Using Lemma 5 and the change of the variable $x = ta + \left( {1 - t} \right)b$, $t \in \left[ {0,1} \right]$ in right hand of (7) along with the fact that $\left| {f^{\prime}} \right|$ is $\eta$-convex imply that

18 $$\begin{aligned} \left| {\frac{f\left( a \right) + f\left( b \right)}{2} - \frac{1}{b - a}\mathop \int \nolimits_{a}^{b} f\left( x \right)dx} \right| & \le \frac{1}{b - a}\left|\mathop \int \nolimits_{a}^{b} (x - \frac{a + b}{2})f^{\prime}\left( x \right)dx\right| \\ & = \frac{{\left( {b - a} \right)}}{2}\left|\mathop \int \nolimits_{0}^{1} (1 - 2t)f^{\prime}\left( {ta + \left( {1 - t} \right)b} \right)dt\right| \\ & \le \frac{{\left( {b - a} \right)}}{2}\mathop \int \nolimits_{0}^{1} \left|\left( {1 - 2t} \right)\right|\left| {f^{\prime}\left( {ta + \left( {1 - t} \right)b} \right)} \right|dt \\ & \le \frac{{\left( {b - a} \right)}}{2}\mathop \int \nolimits_{0}^{1} |\left( {1 - 2t} \right)|\left[ {\left| {f^{\prime}\left( b \right)\left| { + t} \right|\eta \left( {f^{\prime}\left( a \right),f^{\prime}\left( b \right)} \right)} \right|} \right]dt \\ & = \frac{{\left( {b - a} \right)}}{4}\left[ {2\left| {f^{\prime}\left( b \right)\left| + \right|\eta \left( {f^{\prime}\left( a \right),f^{\prime}\left( b \right)} \right)} \right|} \right]. \\ \end{aligned}$$

Similarly if we use the change of variable $x = tb + \left( {1 - t} \right)a$, $t \in \left[ {0,1} \right]$ we have

$$\left| {\frac{f\left( a \right) + f\left( b \right)}{2} - \frac{1}{b - a}\mathop \int \nolimits_{a}^{b} f\left( x \right)dx} \right| \le \frac{{\left( {b - a} \right)}}{4}\left[ {2\left| {f^{\prime}\left( a \right)\left| + \right|\eta \left( {f^{\prime}\left( b \right),f^{\prime}\left( a \right)} \right)} \right|} \right].$$

□

### *Remark 7*

Theorem 6 reduces to Theorem 2.2 in Dragomir and Agarwal (1998), if we consider $\eta \left( {x,y} \right) = x - y$ for all $x,y \in \left[ {a,b} \right]$.

## Conclusions

The convexity of a function is a basis for many inequalities in mathematics and is applicable for nonlinear programming and optimization theory. It should be noticed that in new problems related to convexity, generalized notions about convex functions are required to obtain applicable results. One of this generalizations may be notion of $\eta$-convex functions which can generalizes many inequalities related to convex functions such as the famous Hermite-Hadamard inequality along with estimating the difference between left and middle terms and between right and middle terms of this inequality. Also refinement of Hermite-Hadamard inequality is another application of $\eta$-convex functions.
